# Histological mitotic count is a prognostic marker in vulvar sarcoma: a retrospective case series of 27 patients

**DOI:** 10.1093/jscr/rjaf671

**Published:** 2025-08-30

**Authors:** Zejian Lin, Haifeng Gu, Guochen Liu, Lili Liu, Zhimin Liu, Junyun Li, Xinke Zhang, Hua Tu

**Affiliations:** Department of Gynecologic Oncology, Sun Yat-sen University Cancer Center, State Key Laboratory of Oncology in South China, Collaborative Innovation Center for Cancer Medicine, 651 East Dongfeng Road, Yuexiu District, Guangzhou 510060, Guangdong, China; Department of Gynecologic Oncology, Sun Yat-sen University Cancer Center, State Key Laboratory of Oncology in South China, Collaborative Innovation Center for Cancer Medicine, 651 East Dongfeng Road, Yuexiu District, Guangzhou 510060, Guangdong, China; Department of Gynecologic Oncology, Sun Yat-sen University Cancer Center, State Key Laboratory of Oncology in South China, Collaborative Innovation Center for Cancer Medicine, 651 East Dongfeng Road, Yuexiu District, Guangzhou 510060, Guangdong, China; Department of Pathology, Sun Yat-sen University Cancer Center, State Key Laboratory of Oncology in South China, Collaborative Innovation Center for Cancer Medicine, 651 East Dongfeng Road, Yuexiu District, Guangzhou 510060, Guangdong, China; Department of Gynecologic Oncology, Sun Yat-sen University Cancer Center, State Key Laboratory of Oncology in South China, Collaborative Innovation Center for Cancer Medicine, 651 East Dongfeng Road, Yuexiu District, Guangzhou 510060, Guangdong, China; Department of Radiology, Sun Yat-sen University Cancer Center, State Key Laboratory of Oncology in South China, Collaborative Innovation Center for Cancer Medicine, 651 East Dongfeng Road, Yuexiu District, Guangzhou 510060, Guangdong, China; Department of Pathology, Sun Yat-sen University Cancer Center, State Key Laboratory of Oncology in South China, Collaborative Innovation Center for Cancer Medicine, 651 East Dongfeng Road, Yuexiu District, Guangzhou 510060, Guangdong, China; Department of Gynecologic Oncology, Sun Yat-sen University Cancer Center, State Key Laboratory of Oncology in South China, Collaborative Innovation Center for Cancer Medicine, 651 East Dongfeng Road, Yuexiu District, Guangzhou 510060, Guangdong, China

**Keywords:** vulvar sarcoma, mitotic count, prognosis

## Abstract

This retrospective study analyzed 27 vulvar sarcoma patients treated at one institution (2005–2024). Mitotic count significantly predicted outcomes: patients with ≥20 mitoses/high power field (HPF) (N = 8) showed drastically worse recurrence-free survival (recurrence-free survival; hazard ratio [HR] = 15.48, 95% confidence interval [CI] = 2.67–89.70, *P* < 0.01) and overall survival (HR = 11.97, 95% CI = 1.40–102.22, *P* = 0.02) versus those with 0–9/HPF (N = 12). Despite adjuvant therapy, the ≥20/HPF group experienced high local and distant recurrence rates (both 62.5%). Mitotic count is a robust prognostic indicator, with ≥20 mitoses/HPF indicating poor survival outcomes even after comprehensive treatment.

## Introduction

Vulvar sarcoma is an extremely rare malignancy, accounting for 1%–3% of all vulvar cancers [1]. It is an aggressive malignancy that typically affects women of reproductive age [2]. The histological subtypes of vulvar sarcoma are heterogeneous, including epithelioid sarcoma, leiomyosarcoma, dermatofibrosarcoma protuberans, angiosarcoma, and rhabdomyosarcoma [3, 4]. Unlike other vulvar malignancies, vulvar sarcoma often presents with a benign appearance and non-specific manifestations, which complicates early diagnosis [5].

Because of the extremely low incidence of vulvar sarcoma, most available data are derived from case reports, and no practical guidelines have been established [6, 7]. Therefore, the current clinical management paradigm remains predominantly aligned with the international diagnostic and therapeutic guidelines for systemic soft tissue sarcomas. According to the National Comprehensive Cancer Network guidelines for soft tissue sarcomas, treatment should be based on surgical resection with wide margins to ensure maximal local oncological control [8]. However, the role of lymphadenectomy remains unclear due to limited data. In a review of 28 case reports of vulvar epithelioid sarcoma, lymph node metastasis occurred in 46.2% (6/13) of recurrent cases, whereas none of these patients underwent lymphadenectomy during the initial surgery [9]. Therefore, it is essential to evaluate the therapeutic value of lymphadenectomy in both primary and recurrent vulvar sarcoma.

The biological behavior of vulvar sarcoma is marked by a high propensity for local recurrence and distant metastasis [10]. Consequently, there is a pressing need to identify reliable prognostic factors that can stratify patients into high-risk and low-risk categories, thereby guiding tailored therapeutic approaches. The mitotic count serves as a critical histopathological indicator of biological aggressiveness in sarcomas and has been historically incorporated by the Fédération Nationale des Centres de Lutte Contre le Cancer (FNCLCC) as a pivotal parameter in the grading system for soft tissue sarcomas. In previous studies focusing on low-grade endometrial stromal sarcomas, elevated mitotic activity has been established as a significant independent prognostic determinant [11]. However, the prognostic relevance of mitotic count remains unclear in the context of vulvar sarcomas, necessitating further investigation of its predictive value within this specific histological subset. Researchers have previously conducted a study using the Surveillance, Epidemiology, and End Results database to analyze prognostic factors; however, this study was constrained by a lack of detailed pathological data [12].

In this study, we conducted a retrospective analysis of vulvar sarcoma patients treated at a single institution. Our objectives were to evaluate the clinical features, treatment outcomes, and prognostic factors associated with vulvar sarcoma, with a particular focus on the role of the mitotic count as a potential prognostic predictor.

## Methods

This retrospective study analyzed vulvar cancer patients treated at one institution (2005–2024). Senior pathologists re-evaluated all specimens, confirming diagnoses/mitotic counts. Immunohistochemistry (IHC) was performed routinely; fluorescence in situ hybridization (FISH) was added when indicated. All patients provided informed consent.

Individualized surgical resection ensured adequate margins (width determined by tumor size, location, and suturing feasibility; depth extended to the urogenital diaphragm). Pedicled flap reconstruction was used when necessary. Lymphadenectomy was selective. Due to absent adjuvant therapy guidelines, multidisciplinary team consultations determined postoperative radiotherapy, chemotherapy, or concurrent chemoradiotherapy based on prognostic stratification. Radiation fields were defined by senior oncologists; chemotherapy was tailored.

Clinicopathological/treatment data were extracted from hospital records, follow-ups, and family contacts. Initial treatments of recurrence-referred patients were documented. Recurrence-free survival (RFS: postoperative remission to recurrence/death) and overall survival (OS: hospitalization to cancer-related death/last follow-up) were calculated. Survival curves used Kaplan–Meier methodology with log-rank tests (R v4.4.1; *P* < 0.05 significant).

## Results

**Figure 1:**
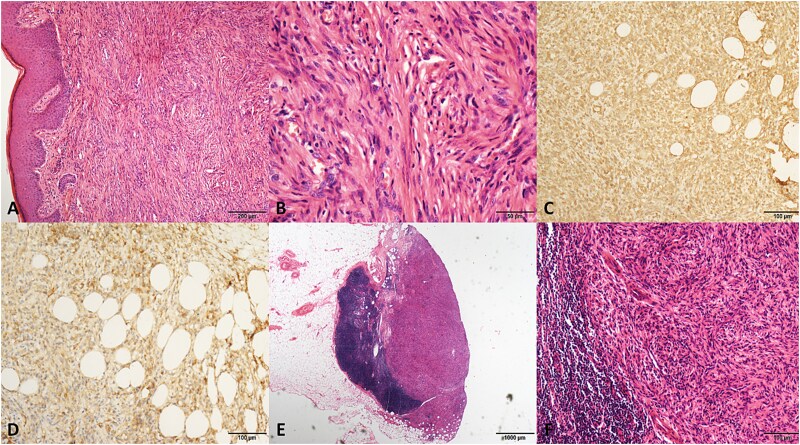
The pathological graphics of No. 2 patient with dermatofibrosaroma protuberans. (A) Spindle-shaped cells in a storiform pattern infiltrating the subdermal tissue (hematoxylin/eosin, original magnification ×100). (B) Original magnification ×400. (C) Positive staining for vimentin (original magnification ×200). (D) Positive staining for CD99 (original magnification ×200). (E) Metastasis of tumor cells in an inguinal lymph node. (hematoxylin/eosin, original magnification ×20). (F) Original magnification ×200.

A total of 27 patients were included. The clinical data are summarized in Table 1 and the results of the IHC examination are summarized in Table 2. All patients were of Chinese ethnicity, with a median age of 42 years at diagnosis (range, 16–71 years). Most patients present with painless subcutaneous masses. The median tumor diameter was 4 cm (range: 1.5–8 cm). Histological subtypes included 14 (51.9%) epithelioid sarcomas, 4 (14.8%) leiomyosarcomas, 4 (14.8%) dermatofibrosarcoma protuberans (DFSP), 3 (11.1%) rhabdomyosarcomas, 1 (3.7%) angiosarcoma, and 1 (3.7%) undifferentiated sarcoma. Some typical pathological images are shown in Fig. 1.

**Table 1:** Clinical-pathological data of the 27 patients with vulvar sarcoma.

**Patient number**	**Age** **(years)**	**Histological type**	**Initial tumor size (cm** ^**2**^ **)**	**Mitoses** **(/10HPF)**	**Tumor status when referred**	**Surgical pattern**	**Lymphatic status**	**Hospital stay (days)**	**Adjuvant treatment**	**RFS** **(months)**	**Additional relapse**	**Site of relapse**	**Outcome**
1	24	LMS	2 × 3	0–9	Resected	WR	N.A.	7	CT	45	Yes	Vulva, lumbar	AWD
2	71	DFSP	3 × 4	≥20	Resected	WR + IL + FT	Positive	22	RT	40	Yes	Pelvis, lung	DOD
3	31	ES	2 × 2	≥20	Relapse (first)	WR + IL	Negative	27	CT	139	Yes		NED
4	28	ES	1.5 × 1.5	≥20	Relapse (second)	WR + IL	Negative	25	CT	2	Yes	Vulva, lumbar	DOD
5	62	ES	5 × 6	≥20	Relapse (second)	WR + FT	N.A.	23	CT	6	Yes	Pelvis	DOD
6	54	DFSP	1.5 × 2	0–9	Resected	WR + IL	Negative	5	None	52	No		NED
7	53	DFSP	2 × 3	0–9	Relapse (third)	WR + FT	N.A.	15	None	107	Yes		NED
8	48	ES	1 × 2	≥20	Relapse (third)	WR + IL + RL + FT	Positive	39	CT + RT	19	Yes	Vulva, liver	DOD
9	46	AS	2 × 3	0–9	Resected	WR	N.A.	20	None	130	No		NED
10	50	LMS	2.5 × 3	0–9	Resected	WR + IL	Negative	25	None	63	No		NED
11	21	DFSP	3 × 3.5	0–9	Resected	WR	N.A.	10	None	114	No		NED
12	16	RMS	4 × 6	0–9	Relapse (first)	WR	N.A.	2	CT + RT	5	Yes	Groin, pelvis	DOD
13	26	RMS	2 × 3	0–9	Resected	WR	N.A.	6	None	47	No		NED
14	45	LMS	2 × 2.5	10–19	Relapse (first)	WR	N.A.	12	RT	87	Yes		NED
15	22	ES	1 × 1.5	0–9	Resected	WR	N.A.	11	None	61	No		NED
16	62	ES	4 × 4.5	≥20	Relapse (first)	WR	N.A.	22	CT + RT	5	Yes	Pelvis, brain	DOD
17	48	LMS	6 × 6.5	≥20	Resected	WR	N.A.	10	CT + RT	28	Yes	Lung, SCN	NED
18	32	RMS	5 × 8	10–19	Resident	WR + IL + FT	Negative	30	CT + RT	6	Yes	Perineum, lung	DOD
19	43	ES	4 × 4	0–9	Resected	WR	N.A.	6	None	74	No		NED
20	65	ES	3 × 4	10–19	Resected	WR + IL	Negative	7	None	70	No		NED
21	37	ES	2 × 3	0–9	Resected	WR	N.A.	10	CT	63	No		NED
22	44	ES	4 × 3	10–19	Resected	WR	N.A.	11	None	29	No		NED
23	62	USTS	8 × 8	≥20	Relapse (first)	WR + FT	Positive	55	CT + RT	37	Yes	Vulva, IGLN	DOD
24	23	ES	N.A.	N.A.	Resected	WR + IL	Negative	14	None	11	No		NED
25	33	ES	3 × 2	0–9	Resected	WR	N.A.	3	None	0	No		NED
26	38	ES	N.A.	N.A.	Resected	WR + IL	Negative	7	None	37	No		NED
27	42	ES	3 × 3.5	N.A.	Resected	WR	N.A.	18	None	39	No		NED

Abbreviations: LMS, leiomyosarcoma; DFSP, dermatofibrosarcoma protuberans; ES, epithelioid sarcoma; AS, angiosarcoma, RMS, rhabdomyosarcoma; USTS, undifferentiated sarcoma; HPF, high power field; WR, wide resection; IL, inguinal lymphadenectomy; RL, retroperitoneal lymphadenectomy; FT, flap transplantation; NA, no assessment; CT, chemotherapy; RT, radiotherapy; NED, no evidence of disease; DOD, died of disease; AWD, alive with disease; IGLN, inguinal lymph nodes; SCN, supraclavicular lymph node.

**Table 2:** The results of IHC examination for 27 patients with vulvar sarcoma.

**Patient number**	**Histological type**	**Expression profile for IHC examination**
1	LMS	SMA(+), HHF35(+), Des(−), MSA(+), Vim(+), CK(+), P53(−), CD34(−), S100(−), Ki67(+)
2	DFSP	Vim (+), CD99 (+), Ki67 (60%+), CD34 (+), Ki67 (+), SMA (−), EMA (−), CK (−)
3	ES	CK(+)、Vimentin(+), S100(−)、HMB45(−), EMA(+),CD34(+), Ki67(15%+), Des(−), HHF35(+), Mel-A(−)
4	ES	Mac387(−), CD68(−), S100(−), HHF35(−), HMB45(−), Melan-A(+/−), CD99(+), Vim(+), Ki67(80% +), CK(+), LCA(−), Des(−), EMA(+), CEA(+/−), Bcl-2(+/−), CK7(+/−)
5	ES	Vim(+), MyoD1(−), inhibin-a(−), L26(−), CD30(−), CK(+), Myogenin(−), S100(−), LCA(−), 34βE12(−), SMA(−), CD99(+/−), P63(−), HMB45(−), Melan-A(−), CD34(−), ALK(+/−), CD56(−), CD3(−), EMA(+), PAX5(−), CD79a(−)
6	DFSP	CD34(+++), STAT6(−), Desmin(−), S-100(−), ER(10%+), Ki-67(2%+)
7	DFSP	CD34(+), S-100(−), SMA(−), Ki67(2%+)
8	ES	CK(+),Vim(+), Desmin(−), EMA(+), CD34(+), CD31(−), HHF35(−), CK7(−), CK5/6(−), P63(−), Myogenin(−), Ki67(30%+)
9	AS	CD 34(+), CD13(+), Fli-1(+), Ki67(10%+)
10	LMS	Vim(+), SMA(+), Des(+), Actin(+), EMA(+), CD34(−), Myoglobin(+/−), CD117(−), HMB45(−), Ki67(15%+)
11	DFSP	CD34(+), Vim(+), SMA(−), HHF35(−), Des(−), S100(−), CD68(−), Ki67(10%+)
12	RMS	Fli-1(−), Myo-G +), des +), HHF35(+), HMB45(−), Ki67(30%+), CD34(−), CD99(−), CK(−), CK-L(−), S100(−), LCA(−), Vim(+)
13	RMS	Vim(+), Myogenin(+), SMA +), Myosin(+), Ki67(40%+), CK(−)
14	LMS	Des(+), SMA(+), Vim(+), HHF35(+), H-Cal(+), Ki67(25%+), CK(−), P53(−)
15	ES	CD34(+), Vim(+), CK(+), P53(+), WT-1(+), EMA(+), Ki67(40%+), MC(−), Des(−), SMA(−), ER(−), PR(−), S100(−), CD99(−), Bcl-2(−), Myoglobin(−), INI1(−), Nestin(+), CD31(−)
16	ES	Vim(+), CK(+), INI-1(−), SNA(+), Ki67(40%+), CD34(−), HMB45(−),MelanA(−), S100(−), CK5/6(−), P63(−),EMA(partial+), CK7(few+), CD56(+), Des(−),
17	LMS	Desmin(+), Caldesmon(+), HHF35(+), Ki67(60%+), Vim(+), Des(+), SMA(+), H-Cal(+),INI1(−), CD10(few+), CD34(−), CD117(−), DOG1(few+), S100(−), CK(−)
18	RMS	Desmin(+), Myogenin(+), MyoD1(+), CD56(+), Syn(few+), CgA(−), NSE(−), CK(−), EMA(−), CD99(−), INI1(+), CD34(−)
19	ES	CK(−), CD34(vascular endothelial+), S100(few+), ALK(−), CD68(few+), SMA(−), Des(−), Ki67(15%+)
20	ES	CK(+), Vim(−), P63(−), CK5/6(−), CK7(−), CK8(+), CR(−), MC(−), EMA(+), SMA(−), Des(−), Calponin(−), β-catenin(−), P40(−), S100(−), HMB45(−), MelanA(−), CgA(−), Syn(−), CD34(+), TFE3(+), WT-1(−), GFAP(−), CD31(−), ERG(−), Fli-1(+), Ki67(5%+), INI1(−)
21	ES	CK (+), EMA(+), HMB45(−), Melan-A(−), S-100(Duo)(−), SMA(+), CD117(−), Calponin(+), CK7(−), p53(partly+), Ki-67(40%+), SALL4(−), Oct-4(−), INI-1(−), Desmin(−), Myogenin(−), CD31(+), CD34(−), ALK(D5F3)(−), ALK(−), CD30(−), ER(−)
22	ES	CK (+), EMA (+), HMB45 (−), SMA (−), Vimentin (+), FLI-1 (+), ERG (+), S100 (−), CD23(−), CD30(−), CD31(+), CD34(−), STAT6 (−), INI-1 (−), F8 (−), SOX10 (−), LCA (−), Desmin (−), ALK (−), Ki-67 (15%+)
23	USTS	CK(AE1/AE3) (−), HMB45 (−), SMA (−), Vimentin (+), CD34 (+), S-100 (−), ALK (−), CD68(KP1) (few-), Melan-A (−), INI-1 (+), CD-10 (−), Ki-67 (80%+)
24	ES	N.A.
25	ES	CK(few+), Vim(−), P16(−), CK5/6(−), CK8/18(+), EMA(+), SMA(few+), Des(−), P40(−), S100(−), HMB45(−), MelanA(−), CD34(−), INI-1(−), Desmin (−), STAT6 (−),CD163 (few+), NF (+), SOX-10 (−), ERG (−), Caldesmon (−), Ki-67 (50%+)
26	ES	CK(AE1/AE3) (−), SMA (−), INI-1 (+), S100(−), Melan-A (−), B-Catenin (+)
27	ES	CK8/18 (−), SMA (−), HMB45 (−), CD99 (−), CD34 (−), S-100 (−), Melan-A (−), Vimentin (+), Desmin (−), Bcl-2 (−), CK-pan (−), ER (−), PR (−), INI-1 (−),Calponin (+), Myogenin (−), MyoD1 (−), ERG (−), Actin (−), Bcl-2 (−), Ki-67 (10%+)

Abbreviations: LMS, leiomyosarcoma; DFSP, dermatofibrosarcoma protuberans; ES, epithelioid sarcoma; AS, angiosarcoma, RMS, rhabdomyosarcoma; USTS, undifferentiated sarcoma.

Eighteen (66.7%) patients were newly diagnosed, while nine (33.3%) patients were referred to our center for recurrent disease. Among the recurrent cases, five (55.6%), two (22.2%), and two (22.2%) patients experienced first, second, and third recurrences, respectively.

All patients underwent wide local excision of vulvar lesions. Inguinal lymphadenectomy was performed concurrently in patients with palpable inguinal lymphadenopathy, whereas laparoscopic pelvic lymphadenectomy was performed in patients with radiologically suspicious pelvic lymph nodes. Intraoperative flap reconstruction was performed based on surgical wound tension assessments.

Nine (33.3%) patients underwent inguinal lymph node resection, and 1 (3.7%) underwent inguinal lymph node resection and pelvic lymph node dissection. Among the 10 patients who underwent lymphadenectomy, three (30.0%) exhibited metastatic lymph nodes, all of which were inguinal lymph node metastases. Despite postoperative adjuvant therapy (chemoradiation in two cases and radiotherapy alone in one case), all three patients developed rapid recurrence and ultimately died from the disease. Adjuvant therapy decisions were determined through multidisciplinary discussions, prioritizing factors such as lymph node metastasis, large tumor size, and high mitotic counts. Of the 12 patients who received adjuvant therapy, 3 (25.0%) received radiotherapy alone, 4 (33.3%) received chemotherapy alone, and 5 (41.7%) received combined chemoradiotherapy. The median radiotherapy dose was 46 Gy (range, 36–60 Gy) and delivered in 18–25 fractions. The chemotherapy regimens included ifosfamide, cisplatin, dacarbazine, epirubicin, and liposomal doxorubicin.

No significant comorbidities were noted, except for Patient No. 6, who had a history of breast cancer (13 years earlier) and endometrial cancer (10 years earlier). Vulvar DFSP was confirmed by FISH, demonstrating a COL1A1-PDGFB fusion.

Excluding three patients with unreported mitotic counts, the remaining cases were stratified into three mitotic activity groups using the FNCLCC grading system: Group A (0–9 mitoses/10 high power field [HPF], n = 12), Group B (10–19 mitoses/10 HPF, n = 4), and Group C (≥20 mitoses/10 HPF, n = 8). The recurrence rates at 5 years were 25.0% (3/12), 50.0% (2/4), and 100.0% (8/8) in Groups A, B, and C, respectively.

**Figure 2:**
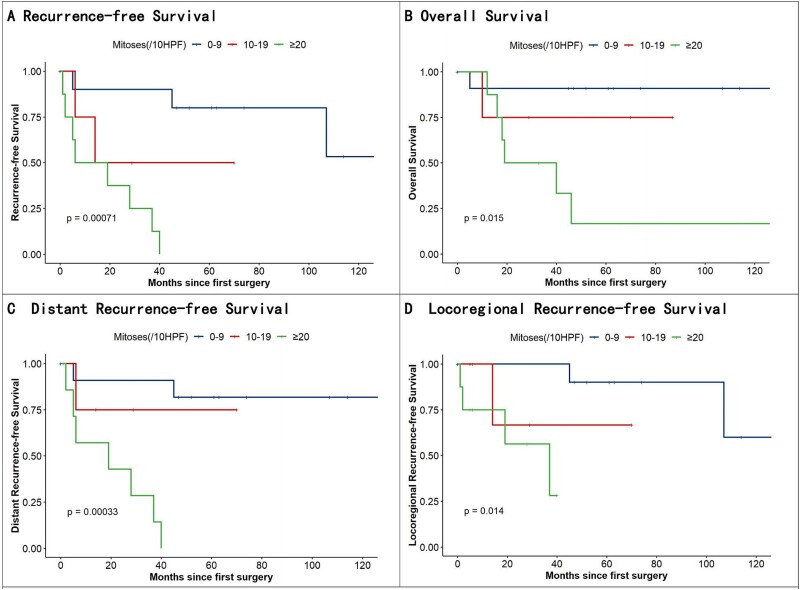
Kaplan–Meier analysis of recurrence-free survival, OS, distant recurrence–free survival, and locoregional recurrence–free survival.

Kaplan–Meier survival curves with Cox regression modeling revealed statistically significant recurrence survival disparities across groups (log-rank *P* < 0.01). Pairwise comparisons using the Cox proportional hazards model identified a marked survival disparity between Groups A and C (*P* < 0.01; hazard ratio = 15.48; 95% confidence interval [CI]: 2.67–89.70). However, no significant difference was observed between groups A and B (*P* = 0.13). Stratified analysis of recurrence patterns showed statistically significant differences in both local (*P* = 0.01) and distant metastatic recurrence rates (*P* < 0.01) between the groups (Fig. 2).

The 5 year mortality rates of groups A, B, and C were 8.3% (1/12), 25% (1/4), and 75% (6/8), respectively, with significant differences in OS (*P* = 0.02). Group A vs. C comparisons remained significant (*P* = 0.02; HR = 11.97; 95% CI: 1.40–102.22), whereas Group A vs. B showed no significance (*P* = 0.37).

During follow-up, among the 12 patients receiving adjuvant therapy, 11 (91.7%) experienced another recurrence, with only one (8.3%) chemotherapy patient remaining recurrence-free. Moreover, the survival outcome was not optimistic. During the follow-up period, seven patients (58.3%) died of the disease.

## Discussion

The present study provides a comprehensive analysis of vulvar sarcoma, a rare and aggressive malignancy for which evidence for clinical practice remains lacking. Our findings underscore the critical prognostic role of mitotic count in this disease, particularly highlighting the dismal outcomes associated with mitotic count ≥20/HPF. Despite multimodal treatment, patients in this subgroup exhibited universally poor survival, with 100% (8/8) recurrence rates and 75% (6/8) mortality at 5 years. These results align with the established role of mitotic activity as a marker of tumor aggressiveness in soft tissue sarcomas and extend its significance to vulvar sarcomas [13], where therapeutic challenges differ substantially from other anatomical sites.

The strong correlation between mitotic count and survival outcomes in groups A and C (RFS: HR = 15.48, *P* < 0.01; OS: HR = 11.97, *P* = 0.02) suggests that cellular proliferation kinetics are central to tumor progression. Mitotic figures reflect genomic instability and uncontrolled cell division, which may drive early metastasis and resistance to conventional therapies [14]. Notably, even patients with mitotic counts ≥20/HPF who received adjuvant therapy, including chemoradiation, experienced high rates of local (62.5%, 5/8) and distant (62.5%, 5/8) recurrence. This observation raises questions regarding the efficacy of current adjuvant regimens for vulvar sarcoma. Although radiotherapy and chemotherapy are the cornerstones of sarcoma management, their utility in vulvar sarcoma may be limited by anatomical constraints and intrinsic tumor resistance mechanisms. The predominance of epithelioid sarcoma in our cohort (51.9%) is noteworthy, as this subtype is known for its propensity for lymphovascular invasion and resistance to traditional chemotherapeutic agents such as doxorubicin [15].

The lack of survival benefits from adjuvant therapy in high-risk patients further emphasizes the need for novel therapeutic strategies. Targeted therapies, such as tyrosine kinase inhibitors or immune checkpoint inhibitors, warrant exploration given their success in other sarcoma subtypes [16]. For example, anti-angiogenic agents or PD-1/PD-L1 inhibitors could be considered, particularly in tumors with specific molecular profiles [17]. The case of Patient No. 6, who developed vulvar dermatofibrosarcoma protuberans with COL1A1-PDGFB fusion, illustrates the potential utility of molecular characterization in guiding therapy [18]. While FISH analysis was performed in only six patients in this study, expanding molecular profiling in future research may identify actionable targets, such as NTRK fusions or TSC1/2 mutations, which are increasingly relevant in sarcoma treatment [19].

Our data revealed persistent challenges. Despite achieving negative margins in all cases, the recurrence rates remained alarmingly high (100%) in the ≥20/HPF group. This suggests that microscopic residual disease or early systemic dissemination may occur, even with optimal local control [20].

The therapeutic role of lymphadenectomy in patients with vulvar sarcoma with lymph node metastasis is limited. In the lymphadenectomy cohort, three patients with metastatic lymph nodes experienced rapid recurrence and mortality, implying that lymph node involvement may signify systemic disease rather than a localized process [21]. This is consistent with prior reports of vulvar epithelioid sarcoma, in which lymph node metastasis was associated with poor outcomes. For patients with lymph node metastasis, exploration of more effective adjuvant treatment strategies is warranted [22].

The heterogeneity of vulvar sarcomas, both histologically and clinically, complicates standardized management. Although epithelioid sarcoma dominated our cohort, the inclusion of leiomyosarcoma, rhabdomyosarcoma, and other subtypes underscores the need for subtype-specific analyses. For instance, angiosarcoma might benefit from anti-angiogenic agents [23]. However, the small sample size of individual subtypes in the present study precludes definitive conclusions, highlighting the necessity for collaborative multi-institutional efforts to pool data and refine treatment algorithms [24].

This study has several limitations. First, its retrospective design introduced a potential selection bias. Second, the small cohort size, though substantial for rare diseases, limits the statistical power for subgroup analyses. Third, the absence of molecular data beyond FISH in most cases restricts our ability to explore the biomarkers or genetic drivers of prognosis.

Future research should prioritize prospective, multicenter collaborations to address these limitations. Integrating next-generation sequencing into clinical workflows could uncover molecular subtypes of vulvar sarcoma with distinct therapeutic vulnerabilities [25]. Additionally, preclinical models, such as patient-derived xenografts or organoids, may help test novel therapies in a controlled setting.

In conclusion, this study established mitotic count as a significant prognostic factor for vulvar sarcoma and identified a subset of patients with ≥20/HPF who had poor prognosis despite aggressive treatment. These findings call for a paradigm shift in managing high-grade vulvar sarcoma, moving beyond conventional therapies toward precision medicine approaches.

## Data Availability

The raw data of this paper are available upon reasonable request to the corresponding author.
